# Puberty and Menapause

**Published:** 1907-08

**Authors:** F. H. Johnson

**Affiliations:** Ensley, Ala.


### PUBERTY AND MENAPAUSE.

BY F. H. JOHNSON, M. D. Ensley, Ala.

There are three distinctive periods in the life of women, two of them being marked by the physiological changes in the reproductive organs, from the berth of the individual to puberty, and from this to the end of her natural existence. One marks the beginning, the other the end of her fruitful period. The gradual development of the organs continue from about the twelfth to the fifteenth year when we have puberty announcing itself by a gradual change taking place in the breast, uterus, ovaries and the external genitals. The breasts increase in size, the hips become large and the contour of the whole body is rounded off. The external genitals and other parts of the body get their growth of hair and menstruation and ovulation are established and the girl becomes more retiring. The menstrual function is not generally established at once, but for the first few months there may be only premonitory symptoms of an uncomfortable nature. There may soon occur a slight discharge of mucus tinged with blood, and later the regular menses will be established. While this is the period which distinctly announces the fact that the girl is capable of reproduction, it is by no means the time advisable for her to become a mother. Statictics plainly show a mortality in those who become mothers before they are developed physically and mentally. The uterus should attain its maximum development, the breast should be fit for nursing, the pelvis should reach a size to admit the passage of a full grown child, the muscle should acquire strength to propel the foetus and the whole system should be endowed with full power of resistance and endurance which seldom occurs before the age of twenty. No doubt we can trace many accidents and bad results in child-bearing to the violation of these laws rather than to the inefficiency of the physician who is in attendance.

Let us now turn our attention to puberty; this marks the first epoich of her existence and in order for these organs to do their work as nature intended without pathological symptoms presenting themselves, the girl must have been surrounded by the proper environment, such as good food, warm clothing and physical exercise in the open air. Irregular menstruation is dependent upon a number of pathological changes which have taken place in the uterus, ovaries, tubes, blood supply and nervous system, or likely all of them. Lack of out-door exercise brings about a lack of muscular development. A failure of proper oxygenation of blood and the development of the organs at puberty is the cause of many of her irregularities, thus we can account for many of the disturbances brought to us for treatments. I am sure we are not careful enough with our girls in educating them in these important rules, and especially are we careless often in finding out the true condition in those brought to us for treatment andadvice. I am sure that the use of the so-called emenogogues are abused, and as I am confident that we do not possess a reliable oxytoxic, so I am that we have no reliable emenogogue. It is only when we have fair development of the individual that we can get relief by these means. A girl with rudimentary uterus or ovaries, anemia or poorly developed nervous system will not respond to these remedies, but must be built up to a point where the organs can act for themselves. A sufficient amount of blood must be obtained to establish a regular periodical flow. These regular periods should continue from puberty to the age of forty-five to forty-eight, occurring every twenty-eight days, except during pregnancy and lactation. The beginning of the second epoch is known as the menopause and is just as physiological as is puberty and if the patient is in good health or condition, should be accompanied with but few disturbances. The same laws that established the flow by reversing themselves should suspend them. The uterus atrophies, the ovaries undergo the same change and we find the blood supply and nervous system undergoing the necessary changes to accommodate themselves to the laws of nature. Unfortunately, due to so many pathological conditions present in the majority of women, we have a long list of nervous symptoms presenting themselves for treatment and here like in the first epoch of our patient. I believe we are not careful enough in making out the true line of the symptoms present. While amenorrhea is the prominent symptom itself at puberty menorrhagia or metrorrhagia are the symptoms at the close of the active period of women. Uterine tonics and general treatment at this stage are generally as unsatisfactory as the emenogagues in the first. We should look for the local cause of trouble and meet it promptly. The idea once so prevelent, that all these symptoms should necessarily be present at this period is untrue. In letting them alone the patient's health becomes depreciated by loss of blood, thus rendering her unable to resist the changes in the nervous system, terminating frequently in ill health or premature death. We should look for the local cause early, such as laceration of the cervix, ulceration and especially carcinoma which so often develops at this period. We often hear the expression even by physicians concerning persons who have long since passed the ovulation period, that the return of the flow is simply a return of the periods. This is unfounded and cannot be based on the physiological action of the pelvic organs. A

person never has a return of their periods after the menopause, without their being some pathological cause for it, and we should search for the cause and if possible meet the indications in each case by direct treatment to what ever lesion is found. The removal of the normal ovaries at this stage to relieve reflex symptoms is a mistake and leaves our patients in a much worse condition than they were before they were operated upon. The nervous symptoms usually increase after the operation and she is more miserable in every way. If it is due to endometritis or to fungus growths in the uterus, it should be dilated and curetted, which will usually result favorably for the patient. Either amputation of the cervix or removal of uterus for carcinomatous conditions should be promptly attended to. Outdoor exercise, general tonic and careful attention to the digestive organs, avoiding constipation and toning up the nervous system, will usually lengthen our patients' lives for usefulness and happiness.

---

Dr. Wm. H. Welch has resigned as a member of the Committee on Legislation of the A. M. A. and Dr. Rodman of Philadelphia has been appointed to succeed him.

---

The corporation of the city of London has decided to present the honorary freedom of the city to Lord Lister in a gold box in recognition of his immense services to humanity. The last time a member of the medical profession was thus honored was over a century ago, when Edward Jenner was given the freedom of London.

---

The Commissioner of Internal Revenue has made a ruling that phsyicians who prescribe for their patients intoxicating liquors not comopunded into medicines and who fill the prescriptions themselves tax of $25 a year.

---

The Society for the Suppression of Unnecessary Noises has secured the passage by the New York city council of a very useful ordinance. It provides that signs shall be placed on the corners of all streets in which a hospital is situated.

